# Crystal structure of (*E*)-1,2-bis­(4-bromo-2,6-di­fluoro­phen­yl)diazene

**DOI:** 10.1107/S2056989015010622

**Published:** 2015-06-10

**Authors:** Johannes Broichhagen, David H. Woodmansee, Dirk Trauner, Peter Mayer

**Affiliations:** a Ludwig-Maximilians-Universität, Department Chemie, Butenandtstrasse 5–13, 81377 München, Germany

**Keywords:** crystal structure

## Abstract

In the crystal, mol­ecules of the centrosymmetric title compound, C_12_H_4_Br_2_F_4_N_2_, are linked into strands along [011] by weak C—H⋯F contacts. Furthermore, the mol­ecules are π–π stacked with perpendicular ring distances of 3.4530 (9) Å.

## Related literature   

For background on azo­benzenes, see: Mitscherlich (1834 ▸); Fehrentz *et al.* (2011 ▸); Banghart *et al.* (2004 ▸); Levitz *et al.* (2013 ▸); Broichhagen *et al.* (2014 ▸); Velema *et al.* (2013 ▸); Bléger *et al.* (2012 ▸). For the synthesis, see: Bléger *et al.* (2012 ▸). For related structures, see: Wragg *et al.* (2011 ▸); Gabe *et al.* (1981 ▸); Crispini *et al.* (1998 ▸); Elder & Vargas-Baca (2012 ▸); Komeyama *et al.* (1973 ▸); Ferguson *et al.* (1998 ▸); Reichenbächer *et al.* (2007 ▸).
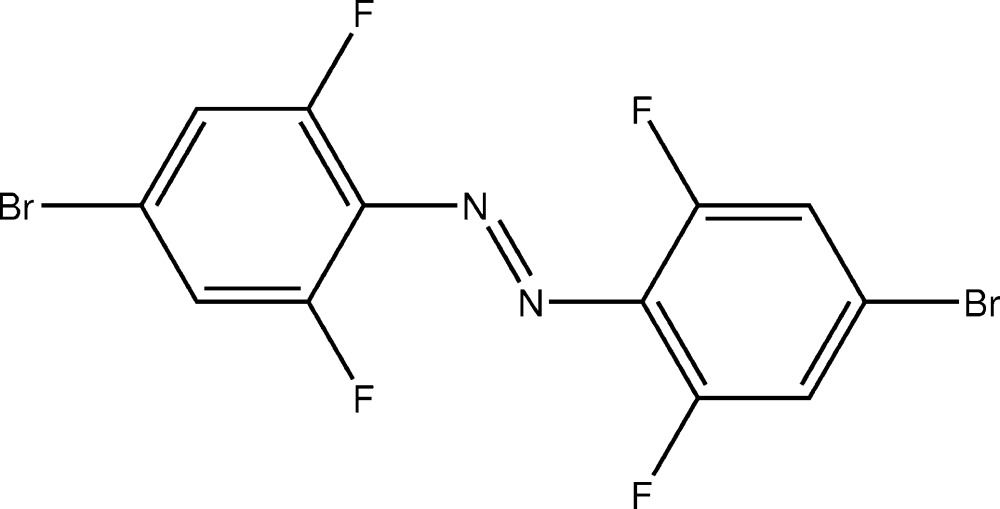


## Experimental   

### Crystal data  

C_12_H_4_Br_2_F_4_N_2_
*M* *_r_* = 411.98
Monoclinic, 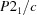

*a* = 10.3274 (5) Å
*b* = 4.5667 (2) Å
*c* = 13.1039 (6) Å
β = 90.340 (3)°
*V* = 618.00 (5) Å^3^
*Z* = 2
Mo *K*α radiation
μ = 6.60 mm^−1^
*T* = 173 K
0.14 × 0.07 × 0.02 mm

### Data collection  

Bruker D8 Quest diffractometer
Absorption correction: multi-scan (*SADABS*; Bruker, 2012 ▸) *T*_min_ = 0.572, *T*_max_ = 0.746
9803 measured reflections
1523 independent reflections
1218 reflections with *I* > 2σ(*I*)
*R*_int_ = 0.051

### Refinement  

*R*[*F* ^2^ > 2σ(*F* ^2^)] = 0.027
*wR*(*F* ^2^) = 0.055
*S* = 1.02
1523 reflections
91 parameters
H-atom parameters constrained
Δρ_max_ = 0.36 e Å^−3^
Δρ_min_ = −0.30 e Å^−3^

### 

Data collection: *Bruker Instrument Service* (Bruker, 2007 ▸); cell refinement: *APEX2* (Bruker, 2007 ▸); data reduction: *SAINT* (Bruker, 2007 ▸); program(s) used to solve structure: *SIR97* (Altomare *et al.*, 1999 ▸); program(s) used to refine structure: *SHELXL2014* (Sheldrick, 2015 ▸); molecular graphics: *ORTEP*-III (Burnett & Johnson, 1996 ▸); software used to prepare material for publication: *PLATON* (Spek, 2009 ▸).

## Supplementary Material

Crystal structure: contains datablock(s) I, global. DOI: 10.1107/S2056989015010622/nr2060sup1.cif

Structure factors: contains datablock(s) I. DOI: 10.1107/S2056989015010622/nr2060Isup2.hkl

**Fig. 1.**
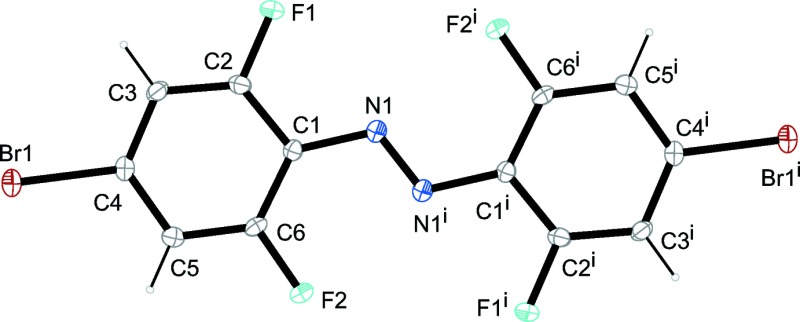
The molecular structure of the title compound, with atom labels and anisotropic displacement ellipsoids (drawn at 30% probability level) for non-H atoms. Symmetry code: (i) 1 - x, 2 - y, 1 - z.

Supporting information file. DOI: 10.1107/S2056989015010622/nr2060Isup3.cml

x y z . DOI: 10.1107/S2056989015010622/nr2060fig1.tif
The mol­ecular structure of the title compound, with atom labels and anisotropic displacement ellipsoids (drawn at 30% probability level) for non-H atoms. Symmetry code: (i) 1 − *x*, 2 − *y*, 1 − *z*.

**Fig. 2.**
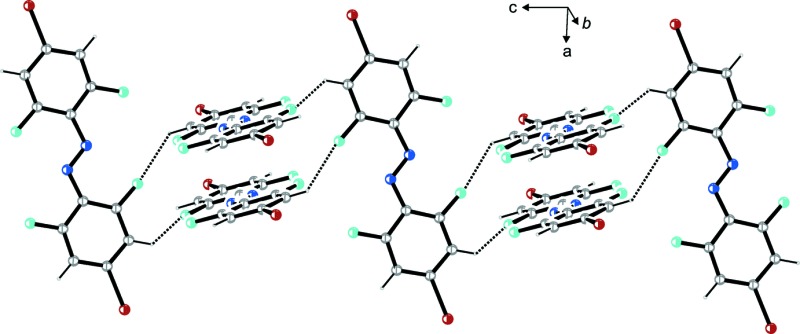
Weak C—H···F contacts (dotted lines) linking the title compound into strands along [011].

. DOI: 10.1107/S2056989015010622/nr2060fig2.tif
Weak C—H⋯F contacts (dotted lines) linking the title compound into strands along [011].

**Fig. 3.**
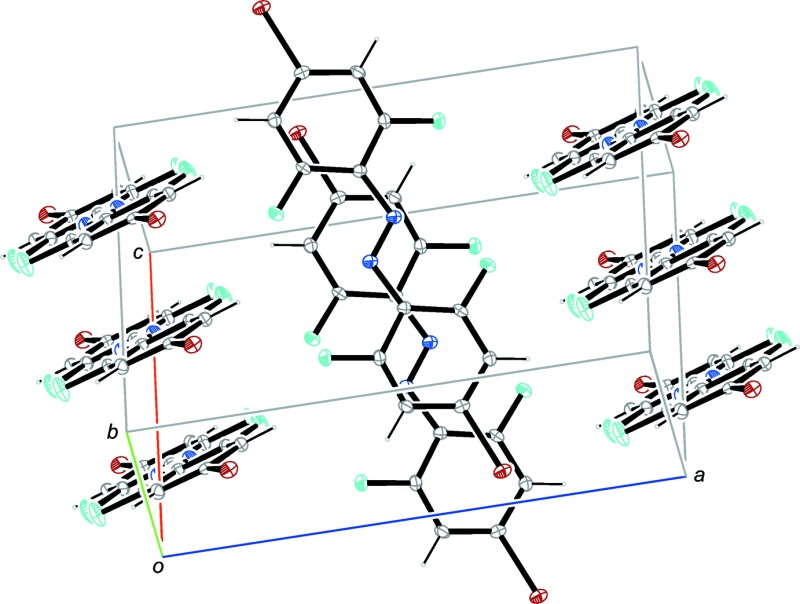
The unit cell of the title compound (displacement ellipsoids drawn at 30% probability level).

. DOI: 10.1107/S2056989015010622/nr2060fig3.tif
The unit cell of the title compound (displacement ellipsoids drawn at 30% probability level).

CCDC reference: 1404445

Additional supporting information:  crystallographic information; 3D view; checkCIF report

## Figures and Tables

**Table 1:** Hydrogen-bond geometry (, )

*D*H*A*	*D*H	H*A*	*D* *A*	*D*H*A*
C5H5F2^i^	0.95	2.53	3.190(3)	126

Symmetry code: (i) 
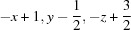
.

## supplementary crystallographic information

### S1. Comment

Azobenzenes, first discovered in 1834 [Mitscherlich (1834)], are experiencing a renaissance in the last decade for the photocontrol of biological function [Fehrentz *et al.* (2011)]. They offer *cis*-/*trans*-isomerism upon irradiation with discrete and orthogonal wavelengths of light. When attached to a pharmacophore, this conformational change has an impact on binding affinities to its biological target. Therefore, a wide variety of transmembrane proteins, such as ion-channels [Banghart *et al.* (2004)] and metabotropic receptors [Levitz *et al.* (2013)], as well as enzymatic activity [Broichhagen *et al.* (2014)] and cell survival [Velema *et al.* (2013)] has been manipulated with azobenzene-based molecular structures. With our ongoing research in photopharmacology, we aimed to synthesize tetrafluoro-azobenzenes, which are characterized by their bistability once isomerized [Bléger *et al.* (2012)]. During our synthetic studies, we obtained (*E*)-1,2-bis(4-bromo-2,6-difluorophenyl)diazene (1) in a crystalline form, which we are reporting herein. This symmetric molecule serves as a precursor for further functionalization and implementation in photopharmacological studies, which will be described in a separate publication.

The molecular structure of the title compound is depicted in Figure 1. There are several structures containing (*E*)-1,2-bis(phenyl)diazene derivatives with at least one halogen substituent in 2-, 4- or 6-position reported in the literature, *e.g.* Wragg *et al.* (2011), Gabe *et al.* (1981), Crispini *et al.* (1998), Elder & Vargas-Baca (2012) and Komeyama *et al.* (1973). Furthermore there are two structures reported which contain the 4-bromo-2,6-difluorophenyl moiety [Ferguson *et al.* (1998), Reichenbächer *et al.* (2007)]. Due to centrosymmetry the phenyl rings are exactly coplanar, however, the entire molecule deviates significantly from exact planarity. The bond of the azo group forms an angle of 4.04 (16)° with the least-square plane of the phenyl ring while it is much larger with 13.21 (12)° in a related structure [Crispini *et al.* (1998)]. The C4–Br1 bond encloses an angle of 2.95 (10)° with the least-square plane of the phenyl ring which is quite close to the angle of 0.2 (3)° in a reported 4-bromo-2,6-difluorophenyl derivative [Ferguson *et al.* (1998)]. The 4-bromo-2,6-difluorophenyl derivative reported of Reichenbächer *et al.* (2007) is suitable for the comparison of bond lengths with the title compound since both compounds have been investigated at 173 K. In the title compound, the C–Br bond distance is 1.888 (2) Å which is almost in the same range of distances found in the reported structure: 1.889 (5), 1.883 (6) and 1.882 (6) Å. The C–F distances are 1.345 (3) and 1.335 (3) Å in the title compound and in the range of 1.336 (7) to 1.353 (7) Å in the related derivative [Reichenbächer *et al.* (2007)].

The packing of the title compound is dominated by weak C–H···F contacts, Br-π contacts and π-stacking. Strands along [011] are formed by C–H···F contacts (see Figure 2 and Table 1 for details). The π-stacking is well visible in Figure 3. The molecules are arranged staggered by what the azo group and the Br substituent of adjacent molecules are located above or below a phenyl ring. The centre of gravity of the phenyl ring (coordinates *x* = 0.28683, *y* = 0.5321, *z* = 0.56133) is in a distance of 3.412 and 3.459 Å from the N-atoms of the azo group (N1^ii^ with ^ii^ = *x*,1 + *y*,*z* and N1^iii^ with ^iii^ = 1 - *x*,2 - *y*,-*z* resp.) and 3.573 (1) Å from an adjacent Br substituent (Br1^iv^ with ^iv^ = *x*,-1 + *y*,*z*). The perpendicular distances of phenyl rings interacting by π-contacts are in a narrow range of 3.4528 (9) and 3.4532 (9) Å with a *Cg*–*Cg* distance of 4.5665 (14) Å. Besides the π contact the Br substituent forms weak contacts to two adjacent Br substituents in a distance of 3.6817 (4) Å each (Br1^v^ and Br1^vi^ with ^v^ = -*x*,1/2 + *y*,1/2 - *z* and ^vi^ = -*x*,-1/2 + *y*,1/2 - *z*).

### S2. Experimental

(*E*)-1,2-bis(4-bromo-2,6-difluorophenyl)diazene (1) was synthesized as reported before and the spectral data matched the previously reported data [Bléger *et al.* (2012)]. Crystals suitable for X-Ray diffractometry were obtained as deep-red needles by slow evaporization from chloroform.

### S3. Refinement

All H atoms were found in difference maps. C-bonded H atoms were positioned in ideal geometry (C—H = 0.95 Å) and treated as riding on their parent atoms [*U*_iso_(H) = 1.2*U*_eq_(C)].

### Crystal data

**Table:**

C_12_H_4_Br_2_F_4_N_2_	*F*(000) = 392
*M**_r_* = 411.98	*D*_x_ = 2.214 (1) Mg m^−^^3^
Monoclinic, *P*2_1_/*c*	Mo *K*α radiation, λ = 0.71073 Å
Hall symbol: -P 2ybc	Cell parameters from 95 reflections
*a* = 10.3274 (5) Å	θ = 5.4–24.6°
*b* = 4.5667 (2) Å	µ = 6.60 mm^−^^1^
*c* = 13.1039 (6) Å	*T* = 173 K
β = 90.340 (3)°	Platelet, orange
*V* = 618.00 (5) Å^3^	0.14 × 0.07 × 0.02 mm
*Z* = 2	

### Data collection

**Table:**

Bruker D8 Quest diffractometer	1523 independent reflections
Radiation source: Microfocus source, Bruker IµS	1218 reflections with *I* > 2σ(*I*)
Focusing mirrors monochromator	*R*_int_ = 0.051
Detector resolution: 10.4167 pixels mm^-1^	θ_max_ = 28.4°, θ_min_ = 3.1°
mix of phi and ω scans	*h* = −13→13
Absorption correction: multi-scan (*SADABS*; Bruker, 2012)	*k* = −6→6
*T*_min_ = 0.572, *T*_max_ = 0.746	*l* = −17→17
9803 measured reflections	

### Refinement

**Table:**

Refinement on *F*^2^	Primary atom site location: structure-invariant direct methods
Least-squares matrix: full	Secondary atom site location: difference Fourier map
*R*[*F*^2^ > 2σ(*F*^2^)] = 0.027	Hydrogen site location: inferred from neighbouring sites
*wR*(*F*^2^) = 0.055	H-atom parameters constrained
*S* = 1.02	*w* = 1/[σ^2^(*F*_o_^2^) + (0.0231*P*)^2^ + 0.2317*P*] where *P* = (*F*_o_^2^ + 2*F*_c_^2^)/3
1523 reflections	(Δ/σ)_max_ < 0.001
91 parameters	Δρ_max_ = 0.36 e Å^−^^3^
0 restraints	Δρ_min_ = −0.30 e Å^−^^3^

### Special details

**Table:**

Geometry. All e.s.d.'s (except the e.s.d. in the dihedral angle between two l.s. planes) are estimated using the full covariance matrix. The cell e.s.d.'s are taken into account individually in the estimation of e.s.d.'s in distances, angles and torsion angles; correlations between e.s.d.'s in cell parameters are only used when they are defined by crystal symmetry. An approximate (isotropic) treatment of cell e.s.d.'s is used for estimating e.s.d.'s involving l.s. planes.
Refinement. Refinement of *F*^2^ against ALL reflections. The weighted *R*-factor *wR* and goodness of fit *S* are based on *F*^2^, conventional *R*-factors *R* are based on *F*, with *F* set to zero for negative *F*^2^. The threshold expression of *F*^2^ > 2*σ*(*F*^2^) is used only for calculating *R*-factors(gt) *etc*. and is not relevant to the choice of reflections for refinement. *R*-factors based on *F*^2^ are statistically about twice as large as those based on *F*, and *R*-factors based on all data will be even larger.

### Fractional atomic coordinates and isotropic or equivalent isotropic displacement parameters (Å^2^)

**Table:**

	*x*	*y*	*z*	*U*_iso_*/*U*_eq_	
C1	0.3771 (2)	0.7379 (5)	0.52174 (17)	0.0188 (5)	
C2	0.2748 (2)	0.6243 (6)	0.46388 (18)	0.0232 (5)	
C3	0.1860 (2)	0.4296 (6)	0.49948 (18)	0.0233 (5)	
H3	0.1179	0.3594	0.4570	0.028*	
C4	0.1983 (2)	0.3376 (5)	0.59963 (18)	0.0197 (5)	
C5	0.2992 (2)	0.4325 (5)	0.66162 (18)	0.0216 (5)	
H5	0.3084	0.3629	0.7296	0.026*	
C6	0.3856 (2)	0.6304 (5)	0.62162 (18)	0.0205 (5)	
N1	0.45691 (19)	0.9452 (5)	0.47266 (15)	0.0234 (4)	
F1	0.26329 (15)	0.7158 (4)	0.36666 (10)	0.0382 (4)	
F2	0.48197 (15)	0.7201 (4)	0.68235 (11)	0.0373 (4)	
Br1	0.07408 (2)	0.08357 (6)	0.656884 (19)	0.02666 (10)	

### Atomic displacement parameters (Å^2^)

**Table:**

	*U*^11^	*U*^22^	*U*^33^	*U*^12^	*U*^13^	*U*^23^
C1	0.0172 (11)	0.0184 (13)	0.0208 (12)	−0.0001 (10)	0.0014 (9)	−0.0017 (10)
C2	0.0266 (13)	0.0257 (14)	0.0173 (12)	−0.0002 (10)	−0.0020 (9)	0.0017 (10)
C3	0.0206 (12)	0.0262 (14)	0.0230 (12)	−0.0035 (11)	−0.0047 (9)	−0.0029 (12)
C4	0.0173 (12)	0.0150 (12)	0.0270 (13)	0.0020 (9)	0.0046 (9)	−0.0014 (10)
C5	0.0239 (12)	0.0215 (13)	0.0193 (11)	0.0016 (11)	−0.0011 (9)	0.0003 (11)
C6	0.0203 (12)	0.0199 (13)	0.0214 (12)	0.0000 (10)	−0.0052 (9)	−0.0049 (10)
N1	0.0221 (11)	0.0239 (11)	0.0243 (11)	−0.0046 (9)	−0.0016 (8)	0.0014 (10)
F1	0.0385 (9)	0.0555 (11)	0.0205 (8)	−0.0178 (8)	−0.0093 (6)	0.0125 (8)
F2	0.0372 (9)	0.0479 (10)	0.0268 (8)	−0.0201 (8)	−0.0142 (7)	0.0079 (8)
Br1	0.02328 (14)	0.02389 (15)	0.03287 (15)	−0.00452 (11)	0.00594 (9)	0.00056 (13)

### Geometric parameters (Å, º)

**Table:**

C1—C2	1.397 (3)	C4—C5	1.387 (3)
C1—C6	1.400 (3)	C4—Br1	1.888 (2)
C1—N1	1.412 (3)	C5—C6	1.376 (3)
C2—F1	1.345 (3)	C5—H5	0.9500
C2—C3	1.362 (3)	C6—F2	1.335 (3)
C3—C4	1.383 (3)	N1—N1^i^	1.244 (4)
C3—H3	0.9500		
			
C2—C1—C6	114.8 (2)	C3—C4—Br1	120.30 (18)
C2—C1—N1	116.3 (2)	C5—C4—Br1	117.97 (18)
C6—C1—N1	128.9 (2)	C6—C5—C4	117.9 (2)
F1—C2—C3	118.1 (2)	C6—C5—H5	121.0
F1—C2—C1	117.5 (2)	C4—C5—H5	121.0
C3—C2—C1	124.4 (2)	F2—C6—C5	117.2 (2)
C2—C3—C4	117.7 (2)	F2—C6—C1	119.4 (2)
C2—C3—H3	121.2	C5—C6—C1	123.3 (2)
C4—C3—H3	121.2	N1^i^—N1—C1	115.1 (2)
C3—C4—C5	121.7 (2)		
			
C6—C1—C2—F1	−178.8 (2)	Br1—C4—C5—C6	−176.42 (17)
N1—C1—C2—F1	1.6 (3)	C4—C5—C6—F2	179.8 (2)
C6—C1—C2—C3	1.7 (4)	C4—C5—C6—C1	−0.5 (4)
N1—C1—C2—C3	−177.9 (2)	C2—C1—C6—F2	178.4 (2)
F1—C2—C3—C4	−179.7 (2)	N1—C1—C6—F2	−2.1 (4)
C1—C2—C3—C4	−0.2 (4)	C2—C1—C6—C5	−1.2 (4)
C2—C3—C4—C5	−1.7 (4)	N1—C1—C6—C5	178.3 (2)
C2—C3—C4—Br1	176.76 (18)	C2—C1—N1—N1^i^	176.3 (3)
C3—C4—C5—C6	2.1 (4)	C6—C1—N1—N1^i^	−3.2 (4)

Symmetry code: (i) −*x*+1, −*y*+2, −*z*+1.

### Hydrogen-bond geometry (Å, º)

**Table:**

*D*—H···*A*	*D*—H	H···*A*	*D*···*A*	*D*—H···*A*
C5—H5···F2^ii^	0.95	2.53	3.190 (3)	126

Symmetry code: (ii) −*x*+1, *y*−1/2, −*z*+3/2.

## References

[bb1] Altomare, A., Burla, M. C., Camalli, M., Cascarano, G. L., Giacovazzo, C., Guagliardi, A., Moliterni, A. G. G., Polidori, G. & Spagna, R. (1999). *J. Appl. Cryst.* **32**, 115–119.

[bb2] Banghart, M., Borges, K., Isacoff, E., Trauner, D. & Kramer, R. H. (2004). *Nat. Neurosci.* **7**, 1381–1386. 10.1038/nn1356 PMC1447674 15558062

[bb3] Bléger, D., Schwarz, J., Brouwer, A. M. & Hecht, S. (2012). *J. Am. Chem. Soc.* **134**, 20597–20600. 10.1021/ja310323y 23236950

[bb4] Broichhagen, J., Jurastow, I., Iwan, K., Kummer, W. & Trauner, D. (2014). *Angew. Chem. Int. Ed.* **53**, 7657–7660. 10.1002/anie.201403666 24895330

[bb5] Bruker (2007). *APEX2*, *Bruker Instrument Service* and *SAINT*. Bruker AXS Inc., Madison, Wisconsin, USA.

[bb6] Bruker (2012). *SADABS*. Bruker AXS Inc., Madison, Wisconsin, USA.

[bb7] Burnett, M. N. & Johnson, C. K. (1996). *ORTEP-III*. Report ORNL-6895. Oak Ridge National Laboratory, Tennessee, USA.

[bb8] Crispini, A., Ghedini, M. & Pucci, D. (1998). *Acta Cryst.* C**54**, 1869–1871.

[bb9] Elder, P. J. W. & Vargas-Baca, I. (2012). *Acta Cryst.* E**68**, o3127. 10.1107/S1600536812040718 PMC3515232 23284452

[bb10] Fehrentz, T., Schönberger, M. & Trauner, D. (2011). *Angew. Chem. Int. Ed.* **50**, 12156–12182. 10.1002/anie.201103236 22109984

[bb11] Ferguson, G., Low, J. N., Penner, G. H. & Wardell, J. L. (1998). *Acta Cryst.* C**54**, 1974–1977.

[bb12] Gabe, E. J., Wang, Y. & Le Page, Y. (1981). *Acta Cryst.* B**37**, 980–981.

[bb13] Komeyama, M., Yamamoto, S., Nishimura, N. & Hasegawa, S. (1973). *Bull. Chem. Soc. Jpn*, **46**, 2606–2607.

[bb14] Levitz, J., Pantoja, C., Gaub, B., Janovjak, H., Reiner, A., Hoagland, A., Schoppik, D., Kane, B., Stawski, P., Schier, A. F., Trauner, D. & Isacoff, E. Y. (2013). *Nat. Neurosci.* **16**, 507–516. 10.1038/nn.3346 PMC3681425 23455609

[bb15] Mitscherlich, E. (1834). *Annalen der Physik und Chemie*, **XXXII**, 224.

[bb16] Reichenbächer, K., Neels, A., Stoeckli-Evans, H., Balasubramaniyan, P., Müller, K., Matsuo, Y., Nakamura, E., Weber, E. & Hulliger, J. (2007). *Cryst. Growth Des.* **7**, 1399–1405.

[bb17] Sheldrick, G. M. (2015). *Acta Cryst.* A**71**, 3–8.

[bb18] Spek, A. L. (2009). *Acta Cryst.* D**65**, 148–155. 10.1107/S090744490804362X PMC2631630 19171970

[bb19] Velema, W. A., van der Berg, J. P., Hansen, M. J., Szymanski, W., Driessen, A. J. & Feringa, B. L. (2013). *Nat. Chem.* **5**, 924–928. 10.1038/nchem.1750 24153369

[bb20] Wragg, D. S., Ahmed, M. A. K., Nilsen, O. & Fjellvåg, H. (2011). *Acta Cryst.* E**67**, o2326. 10.1107/S1600536811032119 PMC3200926 22058951

