# Supplementary figures and images for: Crystal structure of (E)-1,2-bis­(4-bromo-2,6-di­fluoro­phen­yl)diazene
Source: Acta Crystallogr E Crystallogr Commun. 2015 Jun 10;71(Pt 7):o459–60. doi: 10.1107/S2056989015010622 (PMC4518941; doi:10.1107/S2056989015010622)

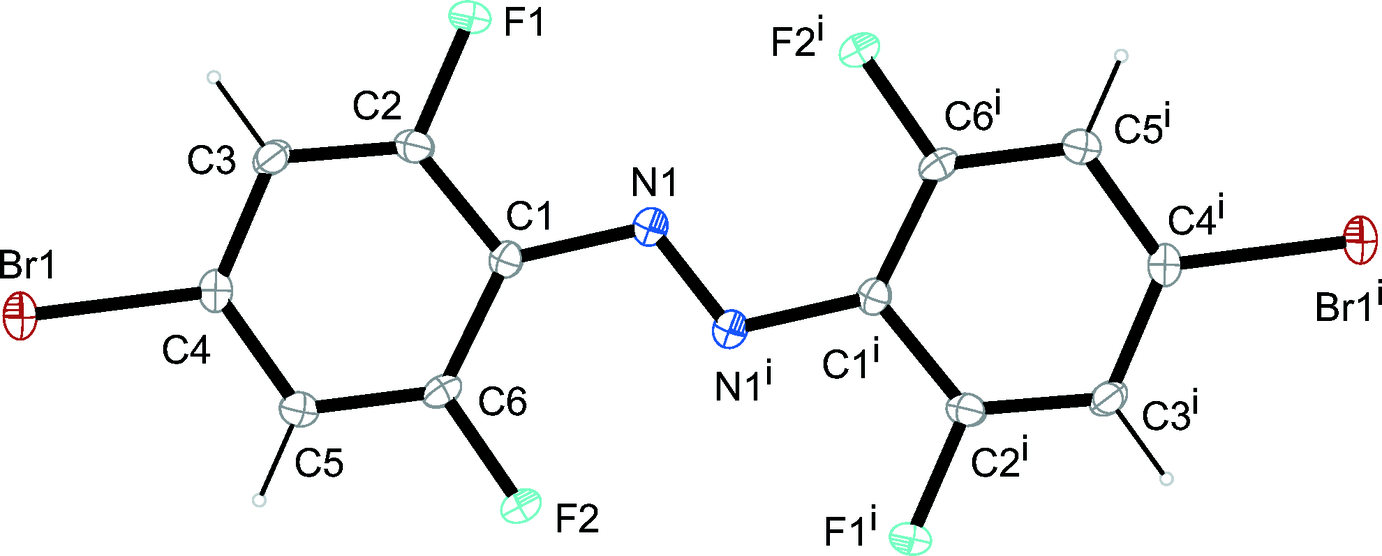

Supplement: Supplementary file 4 [file e-71-0o459-fig1.tif]

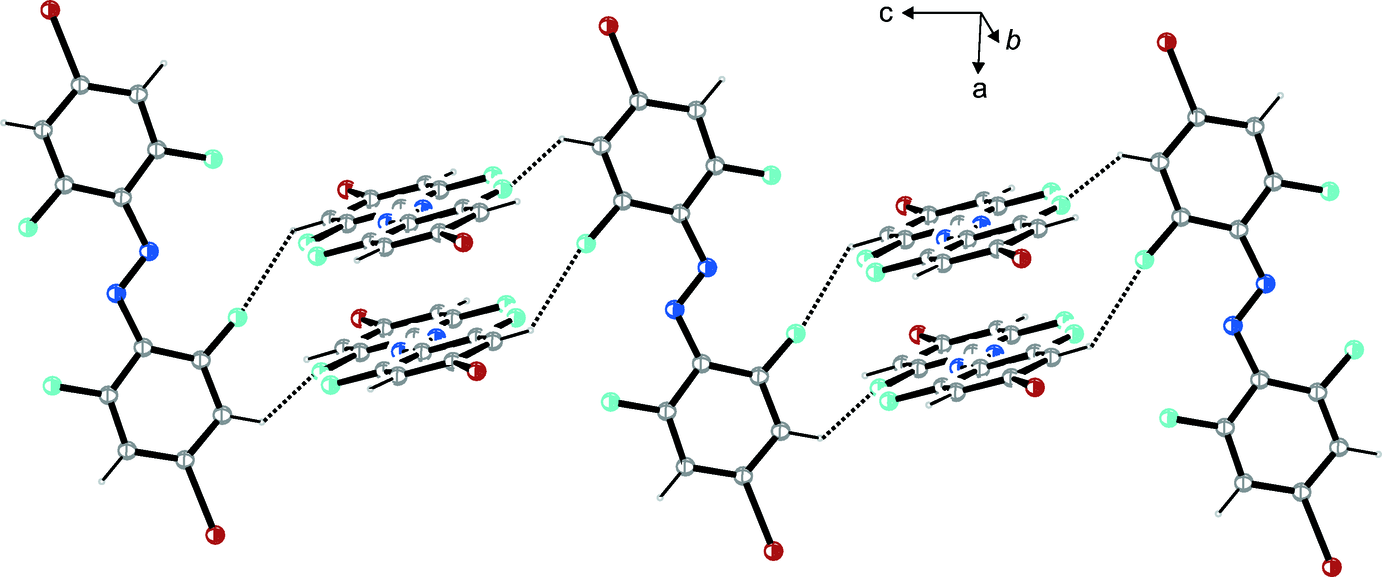

Supplement: Supplementary file 5 [file e-71-0o459-fig2.tif]

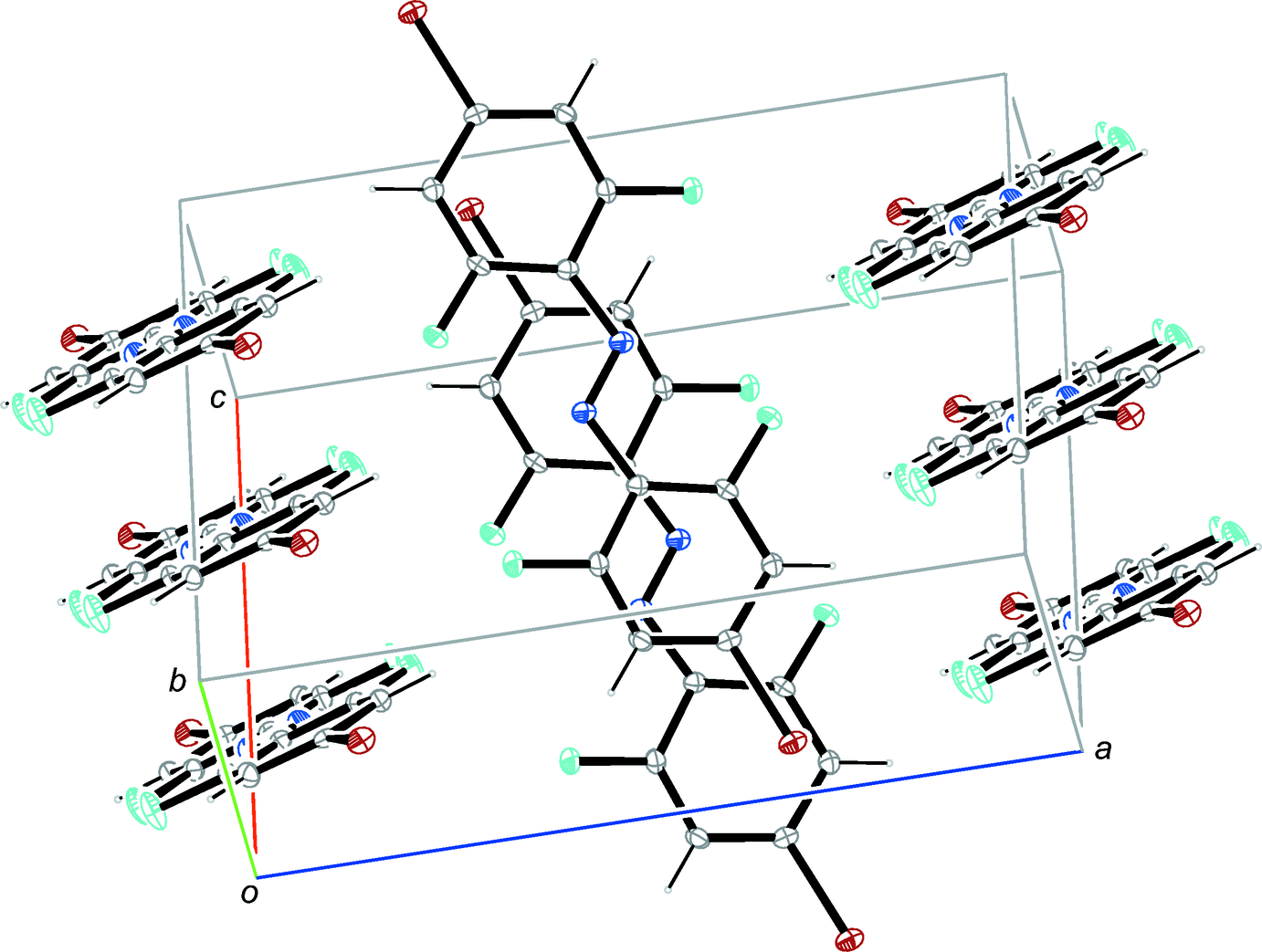

Supplement: Supplementary file 6 [file e-71-0o459-fig3.tif]
